# Lack of collagen XV is protective after ischemic stroke in mice

**DOI:** 10.1038/cddis.2016.456

**Published:** 2017-01-12

**Authors:** Hiramani Dhungana, Mikko T Huuskonen, Taina Pihlajaniemi, Ritva Heljasvaara, Denis Vivien, Katja M Kanninen, Tarja Malm, Jari Koistinaho, Sighild Lemarchant

**Affiliations:** 1 Department of Neurobiology, A. I. Virtanen Institute for Molecular Sciences, Biocenter Kuopio, University of Eastern Finland, Kuopio, Finland; 2 Oulu Center for Cell-Matrix Research, Biocenter Oulu and Faculty of Biochemistry and Molecular Medicine, University of Oulu, Oulu, Finland; 3 Centre for Cancer Biomarkers CCBIO, Department of Biomedicine, University of Bergen, Bergen, Norway; 4 Normandie Univ, UNICAEN, INSERM U919, Serine Proteases and Pathophysiology of the neurovascular Unit, Cyceron, Caen, France; 5 Department of Clinical Research, Caen University Hospital, Avenue de la côte de Nacre, Caen, France

## Abstract

Collagens are key structural components of basement membranes, providing a scaffold for other components or adhering cells. Collagens and collagen-derived active fragments contribute to biological activities such as cell growth, differentiation and migration. Here, we report that collagen XV knock-out (ColXV KO) mice are resistant to experimental ischemic stroke. Interestingly, the infarcts of ColXV KO mice were as small as those of wild-type (WT) mice thrombolysed with recombinant tissue plasminogen activator (rtPA), the actual treatment for ischemic stroke. Importantly, there were no differences in the architecture of cerebrovascular anatomy between WT and ColXV KO mice. We found a twofold increase of the most potent pro-angiogenic factor, type A vascular growth endothelial factor (VEGF-A) in the ipsilateral cortex of rtPA-treated ischemic WT mice compared with untreated ischemic and sham-operated counterparts. A similar increase of VEGF-A was also found in both rtPA and untreated ischemic ColXV KO mice compared with sham ColXV KO mice. Finally, we evidenced that the levels of ColXV were increased in the plasma of WT mice treated with rtPA compared with untreated ischemic counterparts. Altogether, this study indicates that the lack ColXV is protective after stroke and that the degradation of endothelial ColXV may contribute to the beneficial effect of rtPA after ischemic stroke. The neuroprotection observed in ColXV KO mice may be attributed to the increased VEGF-A production following stroke in the ischemic territory.

Stroke is a leading cause of death and long-term disability worldwide.^1^ Ischemic strokes represent 80% of cerebral strokes and result from the obstruction of a major cerebral artery by a thrombus or an embolus which reduces the blood flow in downstream targeted brain regions, leading to brain damage. The establishment of the primary ischemic lesion is followed by a series of secondary events that worsens tissue damage, including vascular, cellular and molecular events.^2^ Nowadays, the only treatment for ischemic stroke is reperfusion with intravenous administration of recombinant tissue plasminogen activator (rtPA; Alteplase) within a strict time frame up to 4.5 h, leading to an improved functional recovery and reduced neurological deficits.^3^ Additionally to its narrowed therapeutic window, rtPA also increases the risk of intracerebral hemorrhagic transformations and is not efficient at degrading platelet-rich thrombi. Taking together all the limitations for the use of rtPA, only 5% of ischemic stroke patients are eligible for rtPA-induced thrombolysis.^4^ Therefore, it is essential to better understand stroke pathophysiology in order to find safer and more efficient approaches for therapy.

Collagens are well-known to be critical extracellular components in vascular stability and functions. Some collagens such as ColXV, ColXVIII and ColXIX have also been shown to be essential for motor axon guidance and neuromuscular development.^5, 6, 7^ The structurally homologous ColXV and ColXVIII constitute the so called multiplexin family of non-fibrillar collagens, characterized by multiple triple helix interruptions and similar non-collagenous sequences.^8^ Nevertheless, they differ in their functional properties and expression patterns and for example, contrary to ColXVIII, ColXV predominantly carries chondroitin sulfate chains and was therefore classified as a chondroitin sulfate proteoglycan (CSPG).^9^ It is mainly produced by skeletal and cardiac muscle, and endothelial cells, occurring at the basement membranes adjacent to these cells. ColXV knock-out (KO) mice suffer from mild skeletal and cardiac myopathy^10, 11^ and defective myelination of peripheral nerves.^12^

In this study, we report for the first time the neuroprotective effect of ColXV deficiency in mice suffering from ischemic stroke. Accordingly, we found an increase of type A vascular endothelial growth factor (VEGF-A) in the ischemic cortex of ColXV KO mice. Additionally, we showed that rtPA increased the presence of unbound ColXV in the plasma of wild-type (WT) mice after stroke.

## Results

### Lack of collagen XV is protective after thromboembolic stroke

**Figure 1:**
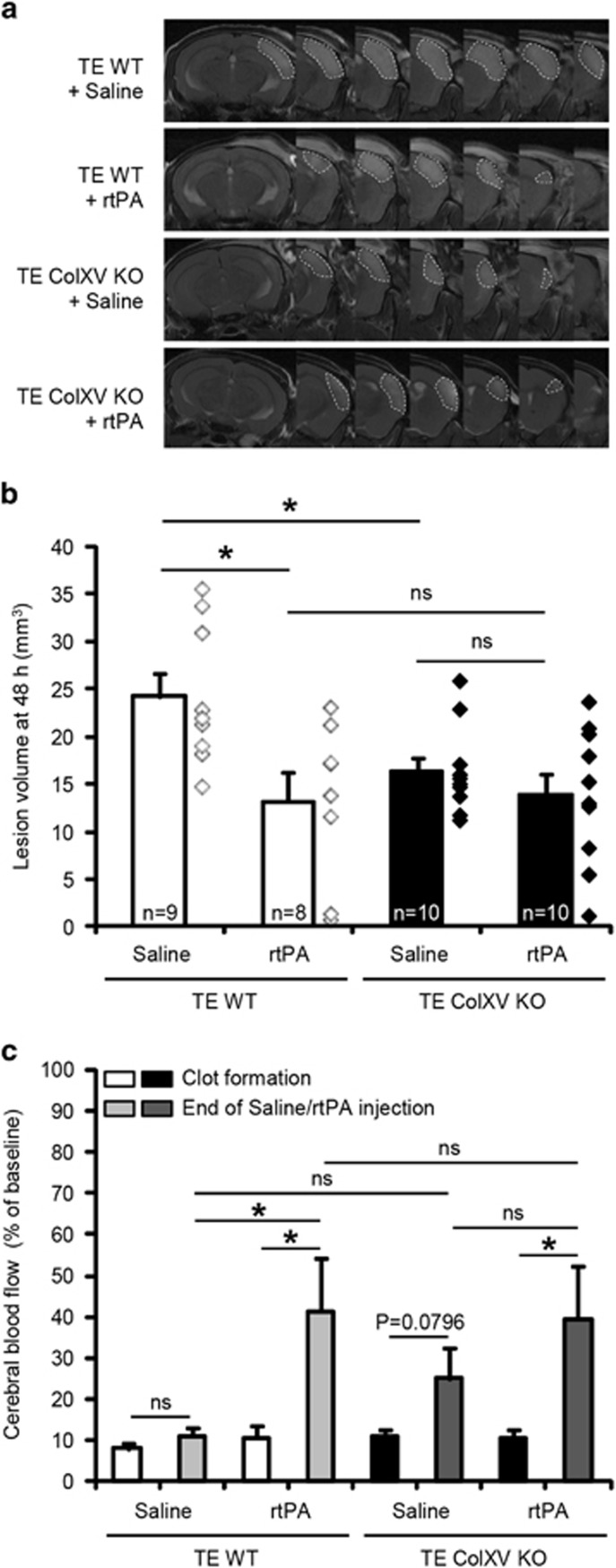
Lack of ColXV is beneficial after thromboembolic stroke. (**a**) Representative T2-weighted MR images of WT and ColXV KO mice 48 h after thromboembolic stroke, reperfused or not with rtPA (10 mg kg^−1^) 20 min after thrombin injection. (**b**) The infarct volume was measured 48 h after stroke. Values plotted are mean±S.E.M. Mann–Whitney *U*-tests: *P<0.05 relative to WT+Saline; *N*=8–10 per group. (**c**) Monitoring of cerebral blood flow registered during surgical approach by Doppler flowmetry. No differences in Doppler reduction after thrombin injection (clot formation) or rtPA-induced recanalization (at the end of Saline/rtPA injection) were noticed between WT and ColXV KO mice. Values plotted are mean±S.E.M. Mann–Whitney *U*-tests: *P<0.05; *N*=8–10 per group

WT and ColXV KO mice were subjected to thromboembolic stroke provoked by a local injection of thrombin into the middle cerebral artery as previously described.^13, 14^ Twenty minutes after clot formation, mice were treated with intravenous injections of saline or rtPA (10 mg kg^−1^). Two days after stroke, magnetic resonance imaging (MRI) images revealed that the lesion volume was significantly smaller in ColXV KO mice compared with WT mice (Figures 1a and b: 24.2±2.5 mm^3^ for WT mice and 16.3±1.5 mm^3^ for ColXV KO mice; *P*=0.0179). Interestingly, while early thrombolysis with rtPA was beneficial in WT mice (Figures 1a and b: 13.2±3.0 mm^3^ for WT mice injected with rtPA; *P*=0.0161 compared with WT mice injected with saline), no additional benefit of rtPA to ColXV KO mice was observed (Figures 1a and b: 13.8±2.3 mm^3^ for ColXV KO mice injected with rtPA; *P*>0.05 compared with ColXV KO mice injected with saline). Cerebral blood flow (CBF) was monitored by Doppler flowmetry during the surgery in WT and ColXV KO mice treated or not with rtPA. We evidenced a similar reduction of about 90% of CBF after thrombin injection (clot formation) in WT and ColXV KO mice (Figure 1c: *P*>0.05 between groups after clot formation). While no changes of CBF were evidenced between clot formation and the end of saline injection in untreated WT and ColXV KO mice (Figure 1c: *P*>0.05), thrombolysis with rtPA increased CBF in WT (Figure 1c: 10.8% of initial CBF at clot formation and 41.4% of initial CBF at the end of rtPA injection in WT mice; *P*=0.0352) and ColXV KO mice (Figure 1c: 10.8% of initial CBF at clot formation and 39.5% of initial CBF at the end of rtPA injection in ColXV KO mice; *P*=0.0474).

**Figure 2:**
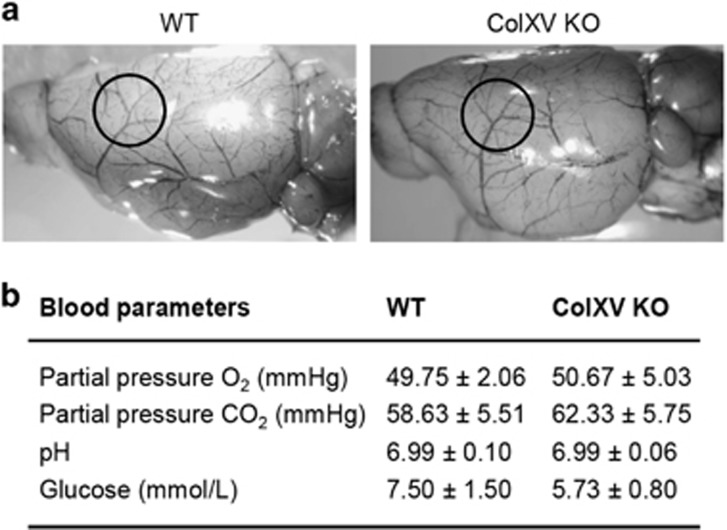
No differences of blood parameters and cerebral vasculatures between WT and ColXV KO mice. (**a**) Measurements of blood parameters in WT (*N*=4) and ColXV KO (*N*=3) mice 20 min after thromboembolic stroke. No significant difference in any of these parameters was noted between WT and ColXV KO mice. (**b**) Cerebral vasculature of WT and ColXV KO mice. Black circles indicate the middle cerebral artery bifurcation

**Figure 3:**
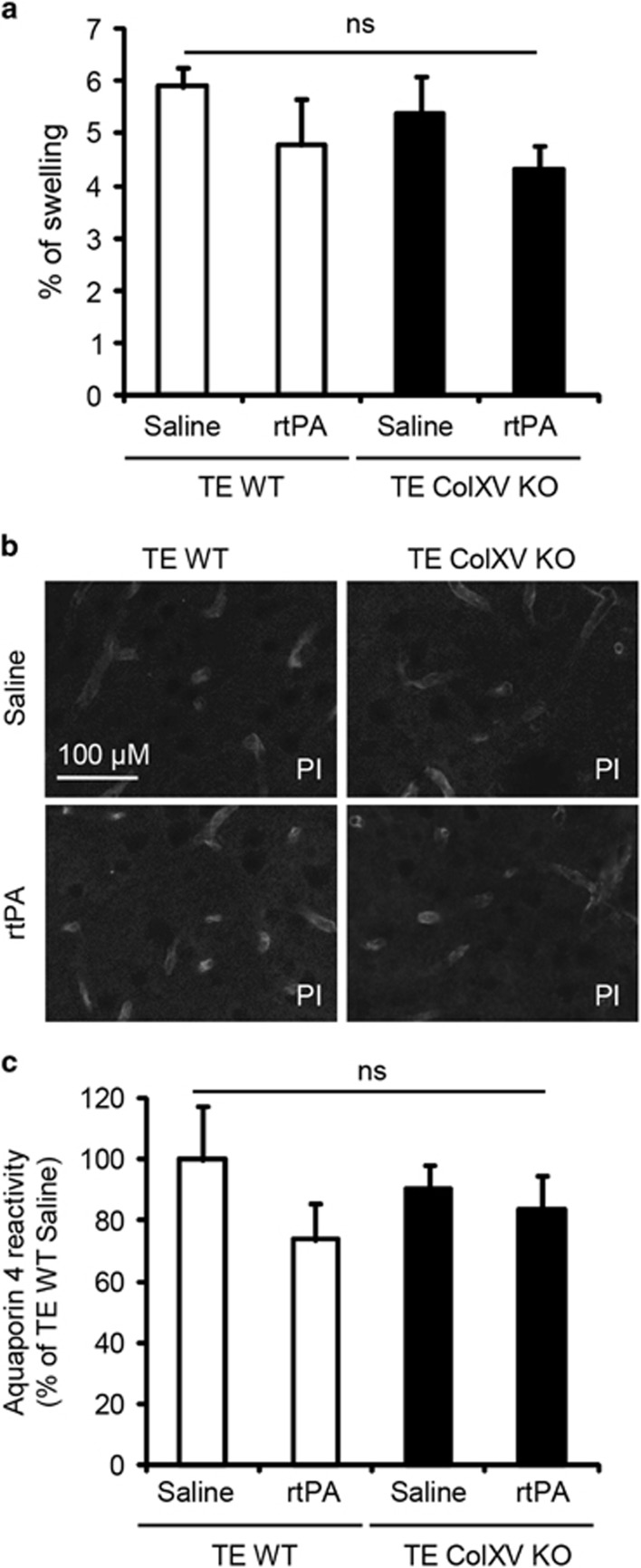
Lack of ColXV does not influence brain edema. (**a**) Brain edema was measured as a % of swelling by using the formula ((V ipsilateral hemisphere−V contralateral hemisphere)/V contralateral hemisphere)*100. Values plotted are mean±S.E.M. Kruskall–Wallis test: *P*>0.05. (**b-c**) Photomicrographs of aquaporin 4 (**b**) and corresponding immunoreactivity quantifications (**c**) in the peri-ischemic area of injured WT and ColXV KO mice thrombolysed or not with rtPA 20 min after thromboembolic (TE) stroke. Values plotted are mean±S.E.M. Kruskall–Wallis test: *P*>0.05; *N*=4–5 per group. PI, peri-ischemic area

We then evaluated a set of features that could account for the protection observed in ColXV KO after ischemia. Importantly, we did not observe any difference between the architecture of cerebral vasculatures in the middle cerebral artery area of WT and ColXV KO healthy mice (Figure 2a). No differences in blood parameters, such as partial pressure O_2_, partial pressure CO_2_, pH or glucose, were found between WT and ColXV KO mice 20 min after stroke (Figure 2b). Brain edema has a central role in the pathophysiology of stroke. Therefore, we looked at brain swelling and aquaporin 4, the well-known glial water channel whose expression is increased after stroke and is extensively described for its contribution to brain edema formation and development.^15^ We did not observe any modification of the percentage of swelling between WT and ColXV KO mice treated or not with rtPA 2 days after stroke (Figure 3a: *P*=0.2444). Similarly, no difference in aquaporin 4 immunoreactivity was found in the peri-ischemic area between WT and ColXV KO mice treated or not with rtPA 3 days after stroke (Figures 3b and c: *P*=0.6393).

### rtPA leads to an increase of collagen XV levels in the plasma of ischemic WT mice

**Figure 4:**
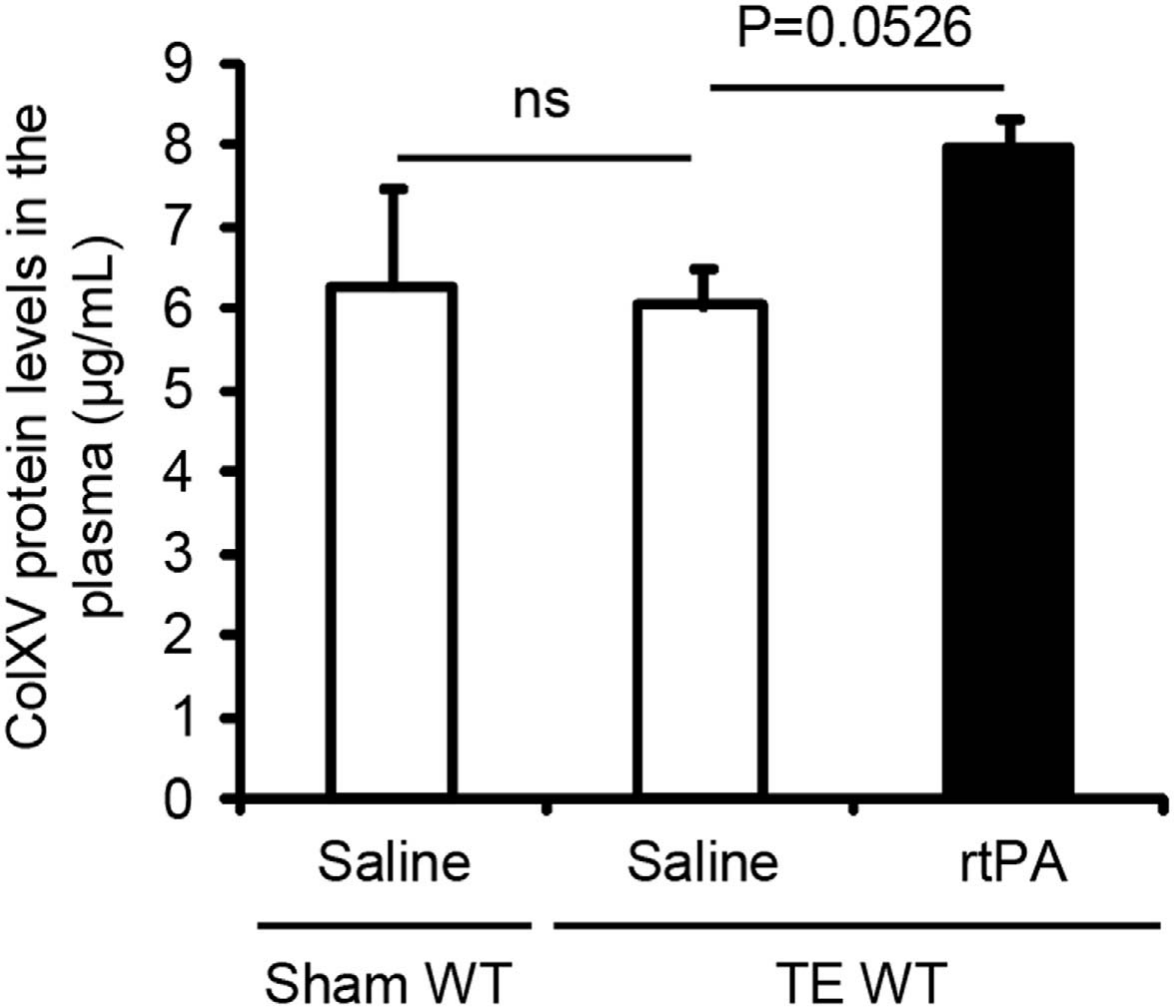
rtPA leads to an increase of ColXV levels in the plasma of WT mice. ELISA for mouse ColXV in the plasma of sham-operated and ischemic WT mice thrombolysed or not with rtPA 20 min after thromboembolic (TE) stroke, at 3 days post injury. Values plotted are mean±S.E.M. Mann–Whitney *U*-tests: *P*=0.0526; *N*=3–5 per group

We then measured the protein levels of ColXV in the plasma of sham-operated and ischemic WT mice treated or not with rtPA 3 days after stroke, by ELISA. Stroke did not influence plasma concentrations of ColXV (Figure 4: *P*=0.6242 between sham-operated and untreated ischemic WT mice). However, rtPA tended to increase ColXV protein levels after ischemia in the plasma of WT mice (Figure 4: +1.93 *μ*g ml^−1^ in ischemic WT mice treated with rtPA compared with untreated ischemic WT mice, *P*=0.0526).

To determine why ColXV KO mice are more resistant to ischemic stroke than WT mice, we then investigated whether the lack of ColXV could influence mechanisms mediating neuroinflammation or neuroprotection.

### rtPA fails to reduce stroke-induced increases of interleukin-6 and chemokine ligand 2 protein levels in ischemic collagen XV KO mice

**Figure 5:**
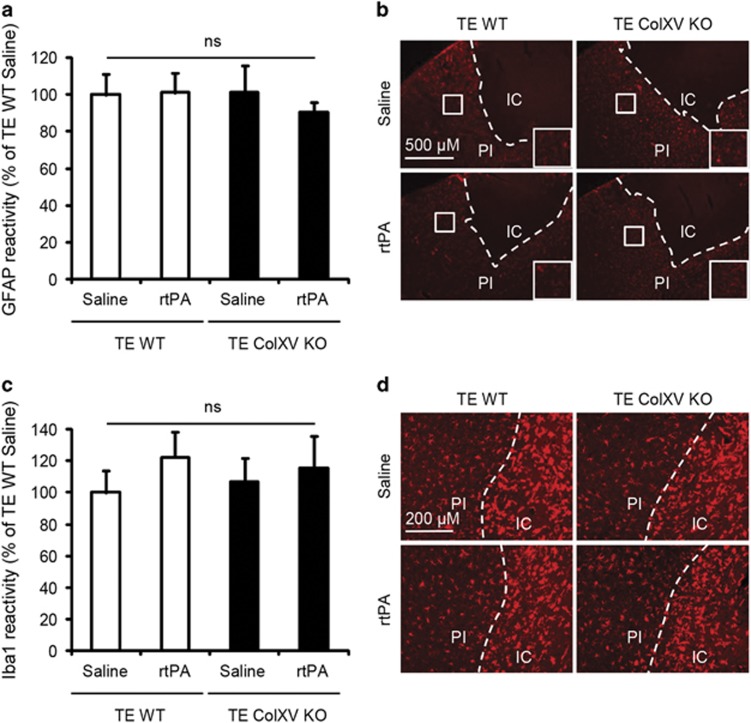
Lack of ColXV does not influence gliosis. Photomicrographs of GFAP (**a**), Iba1 (**c**) and corresponding immunoreactivity quantifications (**b**,**d**) in the peri-ischemic area of injured WT and ColXV KO mice thrombolysed or not with rtPA 20 min after thromboembolic (TE) stroke. Values plotted are mean±S.E.M. Kruskall–Wallis test: *P*>0.05; *N*=4–5 per group. IC, ischemic core; PI, peri-ischemic area

First, we did not observe any changes in astrogliosis or microgliosis between WT and ColXV KO mice treated or not with rtPA 3 days after stroke, measured respectively as GFAP (Figures 5a and b: *P*=0.8330) or Iba1 (Figures 5c and d: *P*=0.6932) reactivity in the peri-ischemic area.

**Figure 6:**
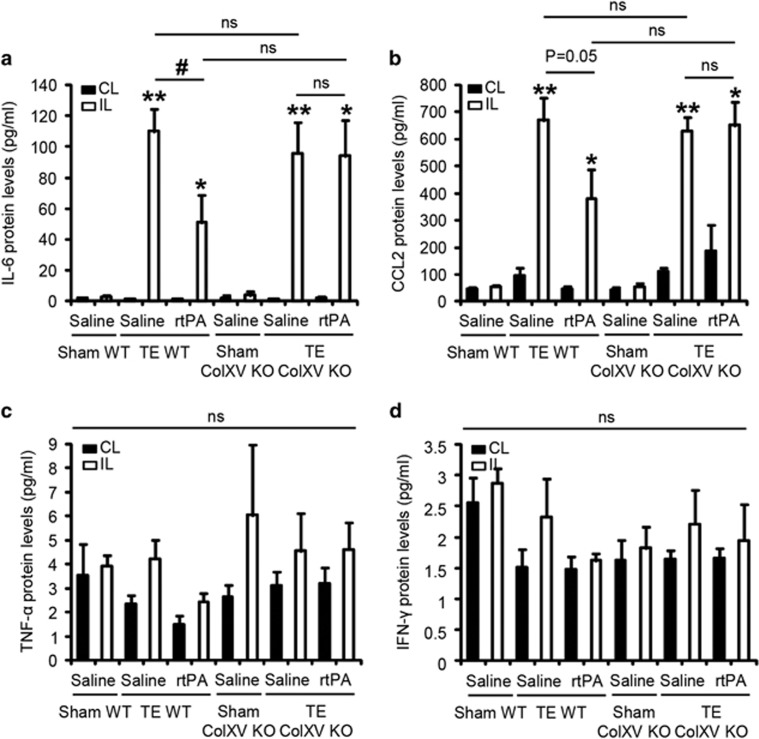
rtPA fails to reduce stroke-induced increases of IL-6 and CCL2 protein levels in the ischemic cortex of ColXV KO mice. IL-6 (**a**), CCL2 (**b**), TNF-*α* (**c**) and IFN-*γ* (**d**) protein levels at 3 days post injury in the contralateral (CL, black bars) and ipsilateral (IL, white bars) cortices of sham-operated and injured WT and ColXV KO mice thrombolysed or not with rtPA 20 min after TE stroke. Values plotted are mean±S.E.M. Kruskal–Wallis: *P*>0.05 for TNF-*α* and IFN-*γ*, *P*<0.0001 for IL-6 and CCL2. Mann–Whitney *U*-tests: *P<0.05, **P<0.01 compared to the contralateral hemisphere, ^#^*P*<0.05 compared to the ipsilateral cortex of saline TE WT; *N* = 4-5 per group

In a parallel cohort of animals, we investigated the expression of cytokines present in protein extracts from the contralateral and ipsilateral cortices of WT and ColXV KO mice treated or not with rtPA 3 days after stroke. Cytometric bead assay revealed a significant increase of interleukin-6 (IL-6) or chemokine ligand 2 (CCL2) protein levels in the ipsilateral cortex of mice suffering from stroke compared with corresponding contralateral cortex or to corresponding sham-operated mice (Figures 6a and b). We observed that thrombolysis with rtPA led to a twofold decrease of stroke-induced IL-6 (Figure 6a: −58.7 pg ml^−1^ in ischemic WT mice treated with rtPA compared with untreated ischemic WT mice, *P*=0.0143) and CCL2 (Figure 6b: −288.7 pg ml^−1^ in ischemic WT mice treated with rtPA compared with untreated ischemic WT mice, *P*=0.0500) increases in the ipsilateral cortex of WT mice. Interestingly, rtPA failed to reduce stroke-induced IL-6 (Figure 6a: *P*=0.6242) and CCL2 (Figure 6b: *P*>0.9999) increases in the ipsilateral cortex of ColXV KO mice. There were no differences of IL-6 (Figure 6c: *P*=0.2506) or CCL2 (Figure 6d: *P*=0.6015) protein levels in the ipsilateral cortex of WT and ColXV KO mice. No modifications of tumor necrosis factor *α* (TNF-*α*) and interferon *γ* (IFN-*γ*) protein levels were found between the groups (Figures 6c and d: *P*=0.3841 for TNF-*α*, *P*=0.3570 for IFN-*γ*). IL-10 and IL-12p70 were not detected in the samples.

### VEGF-A expression is increased in the ischemic cortex of collagen XV KO mice after thromboembolic stroke

**Figure 7:**
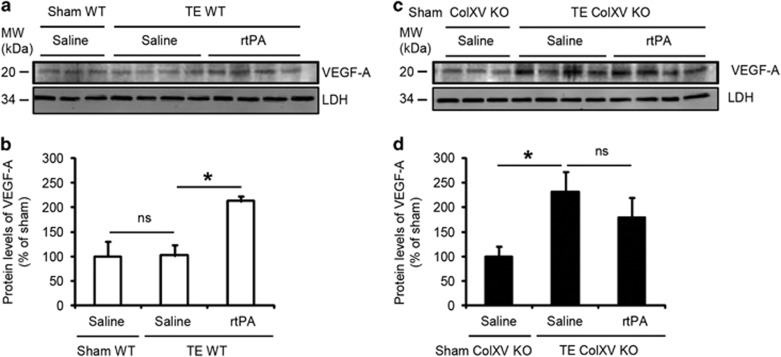
Lack of ColXV increases VEGF-A expression in the ischemic cortex. Immunoblots for VEGF-A (**a** and **c**) and corresponding quantifications (**b** and **d**) in the ipsilateral cortex of sham-operated and injured WT (**a** and **b**) and ColXV KO (**c** and **d**) mice thrombolysed or not with rtPA 20 min after thromboembolic (TE) stroke, at 3 days post injury. Values plotted are mean±S.E.M. Mann–Whitney *U*-tests: *P<0.05; *N*=4

We then studied the expression of VEGF-A, a neuroprotective molecule in the CNS^16^ including after ischemic stroke.^17^ We observed a twofold increase of VEGF-A in the ipsilateral cortex of ischemic WT mice treated with rtPA compared with untreated ischemic WT mice or sham-operated WT counterparts (Figures 7a and b: +110.7% in ischemic WT mice treated with rtPA compared with untreated ischemic WT mice, *P*=0.0209). Interestingly, we observed a similar increase of VEGF-A in ischemic ColXV KO mice treated or not with rtPA compared with sham-operated ColXV KO counterparts (Figures 7c and d: +131.9% in ischemic ColXV KO mice compared with sham-operated ColXV KO counterparts, *P*=0.0209; *P*=0.3865 between ischemic ColXV KO mice treated or not with rtPA).

## Discussion

Collagens, laminins, nidogens and perlecan are major structural proteins of basement membranes, self-assembled with other extracellular matrix (ECM) components, which altogether represent a complex network providing a crucial molecular and physical scaffold for cells.^18^ Here, we investigated for the first time the role of ColXV in the CNS after acute ischemic stroke in mice. Interestingly, we demonstrated that ColXV-deficient mice are more resistant to thromboembolic stroke than WT siblings. Indeed, we observed a 33% decrease in the cortical lesion volume of ColXV-deficient mice compared with WT mice, associated with a twofold increase of VEGF-A in the ischemic cortex. Interestingly, we also showed that rtPA tends to increase the plasma levels of ColXV in ischemic WT mice. Altogether, this study suggests the involvement of ColXV in deleterious pathways occurring during stroke that contribute to worsen brain damage.

ColXV is present in numerous tissues, synthesized by endothelial cells, myoblasts, fibroblasts and epithelial cells, and located at vascular, neuronal, mesenchymal or epithelial basement membrane zones.^19^ Due to its privileged location at the basement membrane zone, ColXV is crucial for the structural integrity of skeletal muscle cells and capillaries.^10, 11^ Indeed, although ColXV KO mice are viable, they develop progressive mild muscle degeneration and abnormal microvessels in heart and skeletal muscles specifically.^10, 11^ Accordingly to the same report,^11^ we did not find any difference in the cerebral vasculature between WT and ColXV KO mice. Surprisingly, one could have expected that ischemic stroke would have worsened brain damage in ColXV KO mice, by weakening the vasculature. On the contrary, ColXV KO mice presented a considerably smaller lesion volume compared with WT mice, with an efficacy similar to the actual treatment for acute ischemic stroke, rtPA.^3^ Unexpectedly, we did not observe any synergic or additional benefit of rtPA in ColXV KO mice. Basement membrane components have the ability to bind other ECM components such as cell surface receptors, cytokines or growth factors.^18^ Therefore, we hypothesized that the basement membranes of ColXV KO mice may have lost their ability to bind and sequester some of these ECM components due to their new structural composition and/or to the absence of ECM component interactions with the chondroitin sulfate chains of ColXV. It could explain VEGF-A over-expression in ischemic ColXV KO mice. Similarly, it would explain the finding that rtPA failed to decrease stroke-induced increases of IL-6 and CCL2 protein levels in ischemic ColXV KO mice. This result suggests that the reduced production of these cytokines by rtPA may be triggered by ColXV. The expression of CCL2 and IL-6 are increased centrally and peripherally after stroke in animal models and in human patients.^20, 21, 22, 23^ Therapies aiming the blockade of CCL2 via a gene transfer or a neutralizing antibody were protective in rodent models of transient middle cerebral artery occlusion (tMCAO).^20, 24^ Similarly, CCL2 KO mice had a smaller lesion volume compared with WT mice after experimental stroke associated with a reduced blood–brain barrier leakage, a decreased infiltration of leukocytes and a decreased production of pro-inflammatory cytokines.^25, 26, 27^ On the contrary, transgenic mice overexpressing CCL2 presented a large ischemic lesion compared with WT mice after permanent MCAO (pMCAO).^28^ Contrary to CCL2, no consensus about the effect of IL-6 after stroke was established to explain the discrepancies observed in animal models and in human patients. Although injection of IL-6 was protective after pMCAO in rats,^29^ the lesion volumes of IL-6 KO and WT mice were similar after tMCAO in mice.^30^ Conversely, increased levels of IL-6 in the serum of human patients correlated with more severe neurological outcomes and a poor diagnosis.^22, 23^

The collagen matrix is continuously remodeled by protease-dependent collagen degradation and production of new collagens. In this regard, our results suggest the possible cleavage of endothelial ColXV by rtPA, possibly participating to the rtPA beneficial effects in ischemic WT mice. This hypothesis is comforted by previous reports showing the ability of rtPA to cleave/degrade CSPGs.^31, 32^ Proteolytic cleavages of ColIV, ColXV and ColXVIII produce fragments with anti-angiogenic activities: arrestin, canstatin or tumstatin for ColIV, restin for ColXV and endostatin for ColXVIII.^33, 34^ Increasing post-stroke repair constitutes one direction of interest to establish new effective therapies. In this regard, VEGF-A is of particular interest for its involvement in mechanisms mediating neuroprotection, angiogenesis, neurogenesis, neuronal migration and survival and axon guidance.^16, 17^ Nevertheless, previous studies caution the use of VEGF for stroke therapy at appropriate dose and time post-stroke.^35^ Here, we have focused on short-term time points: MRI was conducted at 2 days post injury, and the mice were sacrificed one day later. Considering the anti-angiogenic effect of restin and the pro-angiogenic effect of VEGF-A, further investigations are now needed to study a long-term time point, to establish whether the increased VEGF-A production observed early after stroke is not transient but promotes long-lasting angiogenesis in ColXV KO mice after ischemic stroke. In line with our study, compelling evidence shows that CSPGs and heparan sulfate proteoglycans (HSPGs) have critical roles in mechanisms mediating neuroprotection and angiogenesis after stroke. Deglycosylation of CSPGs by the bacterial enzyme chondroitinase ABC reduced glial scar formation, promoted axonal regeneration and collateral sprouting, and improved functional outcome after experimental stroke in rodents.^36, 37^ Similarly, the HSPG glycipan also reduced glial scar and improved functional outcome of rats after pMCAO.^36^ Additionally, chondroitinase ABC and glycipan increased the production of neurotrophic factors in primary cultures of cortical neurons (brain-derived neurotrophic factor for chondroitinase ABC and fibroblast-growth factor 2 for glycipan).^36^ The C-terminal protein fragment domain V of the HSPG perlecan reduced the infarct size and enhanced angiogenesis in a VEGF-dependent manner in the peri-ischemic area thereby promoting functional recovery in several rodent models of ischemic stroke.^37, 38^

This study indicates that the lack of ColXV is protective after ischemic stroke. The neuroprotection observed in ColXV-deficient mice may be attributed to the acutely increased production of a neuroprotective molecule, VEGF-A,^16, 17, 39^ following stroke in the ischemic territory. Further studies are now warranted to decipher the mechanism(s) underlying VEGF-A production in ColXV KO mice during stroke, to determine the cellular origin of VEGF-A (endothelial cells? neurons? glia?), and finally, to investigate whether the increased production of VEGF-A in ColXV-deficient mice may increase angiogenesis during the chronic phase of ischemic stroke. This study also highlights that ColXV may represent a substrate for rtPA, the actual treatment of ischemic stroke.

## Materials and methods

### Ethics

Animal experiments were conducted according to the national regulation of the usage and welfare of laboratory animals, approved by the National Animal Experiment Board of Finland and followed the Council of Europe legislation and regulation for animal protection.

### Animals

Adult WT and transgenic female mice lacking in the *α*1 chain of collagen XV (ColXV KO) by site-specific Cre-*loxP-*mediated deletion in embryonic stem cells from C57BL6J background were used in this study.^11^ Transgenic genotypes were identified by PCR amplification of ear DNA a few days after birth and after death to confirm the results of the first genotyping. The mice were housed under controlled temperature, humidity and light conditions (12 h light and dark cycles) with free access to food and water. Animals were housed in groups of up to 5 in cages.

### Thromboembolic stroke model

Nine month-old WT and ColXV KO female mice were anesthetized by 5% isoflurane in 30% O_2_/70% N_2_O and the surgical anesthesia was maintained by 2% isoflurane. Temperature was maintained at 37±1 °C by thermostatically controlled heating blanket (Harvard apparatus; PanLab, Cornella, Spain). In a parallel group, a femoral artery was catheterized for monitoring of blood gases (*p*H, *p*O_2_, *p*CO_2_) 20 min after ischemia. Mice were placed in a stereotaxic frame, the skin between the right eye and the right ear was incised, and the temporal muscle was retracted. A small craniotomy was performed, the dura was excised, and the middle cerebral artery (MCA) was exposed. One *μ*L containing 1 IU purified murine *α*-thrombin (Enzyme Research Laboratories, South Bend, IN, USA) was injected into the MCA using a micropipette to induce the formation of a stable clot *in situ.*^13, 14^ Cerebral blood velocity in the MCA territory was followed during the surgery by laser Doppler (Moor Instruments, Axminster, UK) to confirm the drop of the blood flow after the clot formation. Thrombolysis was induced after 30 min of occlusion by injecting 200 *μ*l of rtPA (10 mg/Kg; Actilyse, Boerhinger Ingelheim, Germany) intravenously into the tail vein (10% bolus, 90% perfusion for 40 min). Control mice received 200 *μ*l of saline under the same conditions. Mice were randomized into six groups using GraphPad Quickcalcs (GraphPad Software, La Jolla, CA, USA): Sham-operated WT and ColXV KO mice, untreated thromboembolic (TE) stroked WT and ColXV mice, and rtPA-treated TE stroked WT and ColXV mice.

For the first study, mice were sacrificed 3 days after stroke by terminal perfusion with heparinized saline, brains were dissected and the contralateral and ipsilateral cortices were collected and stored at −70 °C for protein purposes (*N*=4–5 in each group). For the second study, mice were sacrificed 3 days after stroke by terminal perfusion with heparinized saline followed by a perfusion with paraformaldehyde (PFA; as described in the immunohistochemistry section) for staining purposes (*N*=4–5 in each group).

### Magnetic resonance imaging

MRI was performed *in vivo* at 2 days post injury in anesthetized mice to determine the lesion volume using a horizontal 9.4 T Oxford NMR 400 magnet (Oxford instrument PLC, Abington, UK) as previously described.^40^ Multi-slice T2-weighted images were acquired: echo time/repetition time of 40 ms/3000 ms, matrix size 128 × 256, field of view 19.2 × 19.2 mm^2^, slice thickness 0.8 mm and number of slices 12. Images were then analyzed using Aedes software (Kuopio, Finland) under MatLab program (Math-works, Natick, USA).

### Protein extraction

Contralateral and ipsilateral cortices were dissociated in ice-cold TNT buffer (50 mM Tris-HCl pH 7.4; 150 mM NaCl; 0.5% Triton X-100) containing EDTA/EGTA (ethylene diamine/glycol tetraacetic acid, 1 mM), protease (Sigma-Aldrich, St. Louis, MO, USA) and phosphatase (Roche Diagnostics, Mannheim, Germany) inhibitors. Debris were removed by centrifugation (12 000 × *g* at 4 °C, 15 min). Supernatants were stored at −70 °C until further processing. Protein quantification was performed according to the BCA protein method (Pierce, Rockford, USA).

### Western blot

Proteins (5 *μ*g) were resolved on 14% polyacrylamide gel under denaturing conditions and transferred onto a polyvinylidene difluoride membrane. Membranes were blocked with phosphate-buffered saline (PBS) tween (0.2% Tween-20; Sigma-Aldrich) and 5% of milk. Blots were incubated overnight at 4 °C with the mouse anti-VEGF-A (1/500; Santa Cruz Biotechnology, Dallas, TX, USA) primary antibody diluted in PBS–tween containing 5% of milk. After a 2- h incubation at room temperature (RT) with the peroxidase-conjugated anti-mouse secondary antibody (1/5000; GE Healthcare Life Sciences, Uppsala, Sweden), proteins were revealed with an enhanced chemiluminescence ECL-Plus kit immunoblotting detection system (GE Healthcare Life Sciences) and visualized using Storm FluorImager system. Rabbit anti-LDH (1/500; Santa Cruz Biotechnology) was used as a loading control^41, 42^ and visualized by Alexa fluor 647-conjugated anti-rabbit secondary antibody (1/1000; Jackson ImmunoResearch Laboratories, West Grove, PA, USA).

### Immunohistochemistry

Anesthetized mice were perfused with cold heparinized saline, followed by a perfusion with 4% PFA in 0.1 M phosphate buffer (PB) pH 7.4. Brains were collected and rinsed in a PB containing 20% sucrose for cryoprotection for 24 h and then embedded and frozen in OCT (Optimal Cutting Temperature; Sakura Finetek, Tokyo, Japan). Six 20- *μ*m-coronal sections 200 *μ*m apart of each brain were cut on a cryostat (Leica Microsystems, Wetzlar, Germany), collected on poly-lysine glasses (Thermo Scientific, Leicestershire, UK), and stored at −70 °C until analysis. After washing with PB, PBS and PBS–tween (0.05% Tween-20), sections were treated when required with PBS–Triton X-100 (0.4%, Sigma-Aldrich) and unspecific bindings were blocked by 1-h incubation in 10% normal goat serum (NGS, Merck Millipore, Billerica, MA, USA). Incubation with primary antibodies was conducted overnight at RT with dilutions as follows: rabbit anti-GFAP (glial fibrillary acidic protein, 1/200; Dako), rabbit anti-Iba1 (ionized calcium-binding adapter molecule-1, 1/250; Wako Pure Chemical Industries, Tokyo, Japan) or rabbit anti-aquaporin 4 (1/300; Merck Millipore). After washing with PBS–tween, sections were incubated with corresponding fluorescent Alexa fluor −488 or -568-conjugated secondary antibodies (1/200; Life Technologies) for 2 h at RT, then washed again and finally mounted in Vectashield with DAPI (Vector Laboratories, Burlingame, CA, USA). Negative controls for unspecific binding of the secondary antibodies were conducted in parallel sections following the same procedures described above, except the incubation in primary antibodies.

For the analyses, the peri-ischemic area was imaged using 10x magnification on an AX70 microscope (Olympus corporation, Tokyo, Japan) coupled to a digital camera (Color View 12, soft Imaging System, Muenster, Germany) using Soft Imaging software. Immunoreactivity were quantified using Image-Pro Plus software (Media Cybernetics, Rockville, MO, USA) at a pre-defined range, measured as the relative immunoreactive area for GFAP, Iba1or aquaporin 4. Analysis were performed blinded to the study groups (*N*=4–5 in each group).

### ColXV ELISA

Terminal blood samples were collected from the heart into tubes containing 10% vol/vol 3.8% trisodium citrate (pH 5.2 adjusted with citric acid). Plasma samples were collected after two consecutive centrifugations, one for 15 min at 2000 × *g* and one or three min at 12 000 × *g*. ELISA for mouse ColXV was performed according to the manufacturer's instructions (LSBio, Seattle, WA, USA) using plasma samples diluted 1:2.

### Cytokine protein expression

IL-6, CCL2, TNF-*α*, IFN-*γ*, IL-10 and IL-12p70 proteins contained in protein lysates from the contralateral and ipsilateral cortices of sham-operated and ischemic WT and ColXV KO mice treated or not with tPA were measured by using the cytometric bead assay Th1/Th2/Th17 kit (BD Biosciences, Franklin Lakes, NJ, USA) according to the manufacturer's instructions. Data were acquired using FACSCalibur (BD Biosciences, San Jose, CA, USA) and analyzed by FCAP Array software (Soft Flow, St. Louis Park, MN, USA).

### Middle cerebral artery territory

To evaluate the cerebrovascular anatomy of WT and ColXV KO mice, anesthetized mice were perfused with saline as described in the IHC section, then with a diluted India blue ink dye solution and finally with PFA. The brains were dissected out and the images were taken using stereomicroscope (Ziess, Oberkochen, Germany) to determine the difference in vasculature around the MCA territory.

### Statistical analyses

The data are expressed as mean±S.E.M. An alpha level of *P*<0.05 was used for determination of significance in all statistical tests. Statistical analyses were performed with the Statview software package (v5.0). Kruskal–Wallis test was used for intergroup multiple comparisons. In significant cases, Mann–Whitney *U*-test was applied as *post hoc* test.
